# Outcomes of Left Bundle Branch Area Pacing in Heart Failure Patients: A Systematic Review

**DOI:** 10.1111/anec.70158

**Published:** 2026-02-02

**Authors:** Hina Ahmed Siddiqui, Mounika Kotte, Hilana Soliman Omar, Saad Manzoor, Zohaib Qasim, Fnu Abdullah, Sadia Siddique, Muhammad Bari Hassan, Ali Karim, Hansa Devi, Ekta Rani, Aniket Tara, Payal Bai, Sajid Ali, Hina Kumari, Sitara Jabeen, Vikram Kumar, Abida Perveen

**Affiliations:** ^1^ Department of Medicine Ibn e Seena Hospital Kabul Afghanistan

**Keywords:** heart failure, LBBAP, left bundle branch area pacing, pacingcardiac resynchronization therapy, reduced ejection fraction

## Abstract

**Background:**

Left bundle branch area pacing (LBBAP) is an emerging physiological pacing technique that restores ventricular electrical synchrony by directly engaging the left conduction system. It has been proposed as an alternative to conventional pacing strategies, particularly in heart failure patients with reduced left ventricular ejection fraction (LVEF ≤ 50%).

**Methods:**

A systematic literature search of PubMed, MEDLINE, and Scopus was conducted up to December 2024 in accordance with PRISMA guidelines. Sixteen studies involving 5680 patients were included. Reported outcomes included changes in LVEF, QRS duration (QRSd), hospitalization rates, complications, and mortality. Due to heterogeneity among studies, a qualitative narrative synthesis was performed.

**Results:**

LBBAP was associated with significant improvements in cardiac function, with most studies reporting increased LVEF and marked reductions in QRSd, indicating improved electrical synchrony. Complication rates were low, with rare events such as pneumothorax and lead dislodgement. Heart failure–related hospitalizations were lower with LBBAP compared with biventricular pacing (19.05% vs. 30.00%), while mortality rates remained low across pacing strategies. Overall, LBBAP demonstrated superior electrical resynchronization and favorable clinical outcomes compared with conventional pacing modalities.

**Conclusion:**

LBBAP is a promising pacing strategy that improves electrical synchrony and cardiac function with a favorable short‐ to mid‐term safety profile. Further large‐scale randomized studies are needed to establish its long‐term efficacy and safety.

## Graphical abstract

LBBAP demonstrated significant improvements in cardiac function, with most studies reporting substantial increases in LVEF and reductions in QRSd. LBBAP is a promising alternative to conventional pacing strategies, offering superior electrical and mechanical synchronization, enhanced cardiac function, and better clinical outcomes.

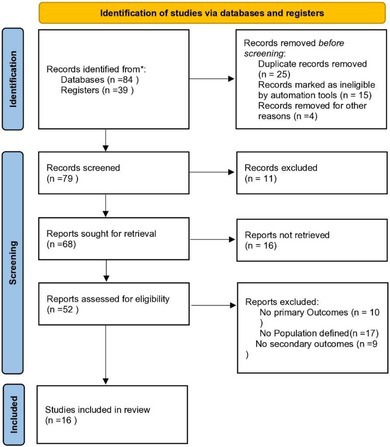

## 1 Introduction

Heart failure (HF) is a prevalent and debilitating condition that results from impaired myocardial function, leading to symptoms such as fatigue, dyspnea, and fluid retention (Sapna et al. 2023). One of the critical indicators of heart failure severity is left ventricular ejection fraction (LVEF), with patients having an LVEF ≤ 50% typically categorized as having reduced ejection fraction heart failure (HFrEF) (Murphy et al. 2020). This group of patients often experiences prolonged QRS duration, indicating electrical dyssynchrony, which exacerbates the disease's progression and worsens clinical outcomes (Hummel et al. 2009).

Cardiac resynchronization therapy (CRT), which involves either biventricular pacing or right ventricular pacing in combination with left ventricular pacing, is an established intervention for improving mechanical synchronization and overall cardiac function in heart failure patients (Jaffe and Morin 2014). However, the traditional approaches, particularly right or biventricular pacing, often fail to fully restore physiological conduction and may lead to adverse effects such as right ventricular pacing‐induced dyssynchrony (Tops et al. 2006). This limitation has led to the exploration of alternative pacing strategies, one of the most promising being Left Bundle Branch Area Pacing (LBBAP) (Liu, Wang, et al. 2021).

LBBAP targets the left bundle branch or its proximal region, offering a more physiological method of pacing that potentially restores normal conduction and improves both electrical and mechanical synchrony (Tun et al. 2023). Early studies suggest that LBBAP may be a more effective and safer alternative compared to conventional pacing strategies, particularly for patients with HFrEF and LVEF ≤ 50%. However, despite these promising outcomes, the overall efficacy and safety of LBBAP in this patient population remain areas of ongoing research (Fu et al. 2022).

This systematic review aims to evaluate the efficacy and safety of LBBAP as a physiological alternative to traditional right and biventricular pacing in heart failure patients with LVEF ≤ 50%, with a focus on its impact on clinical outcomes, electrical and mechanical synchronization, and long‐term safety.

## 2 Methods

### 2.1 Study Design and Guidelines

**FIGURE 1:**
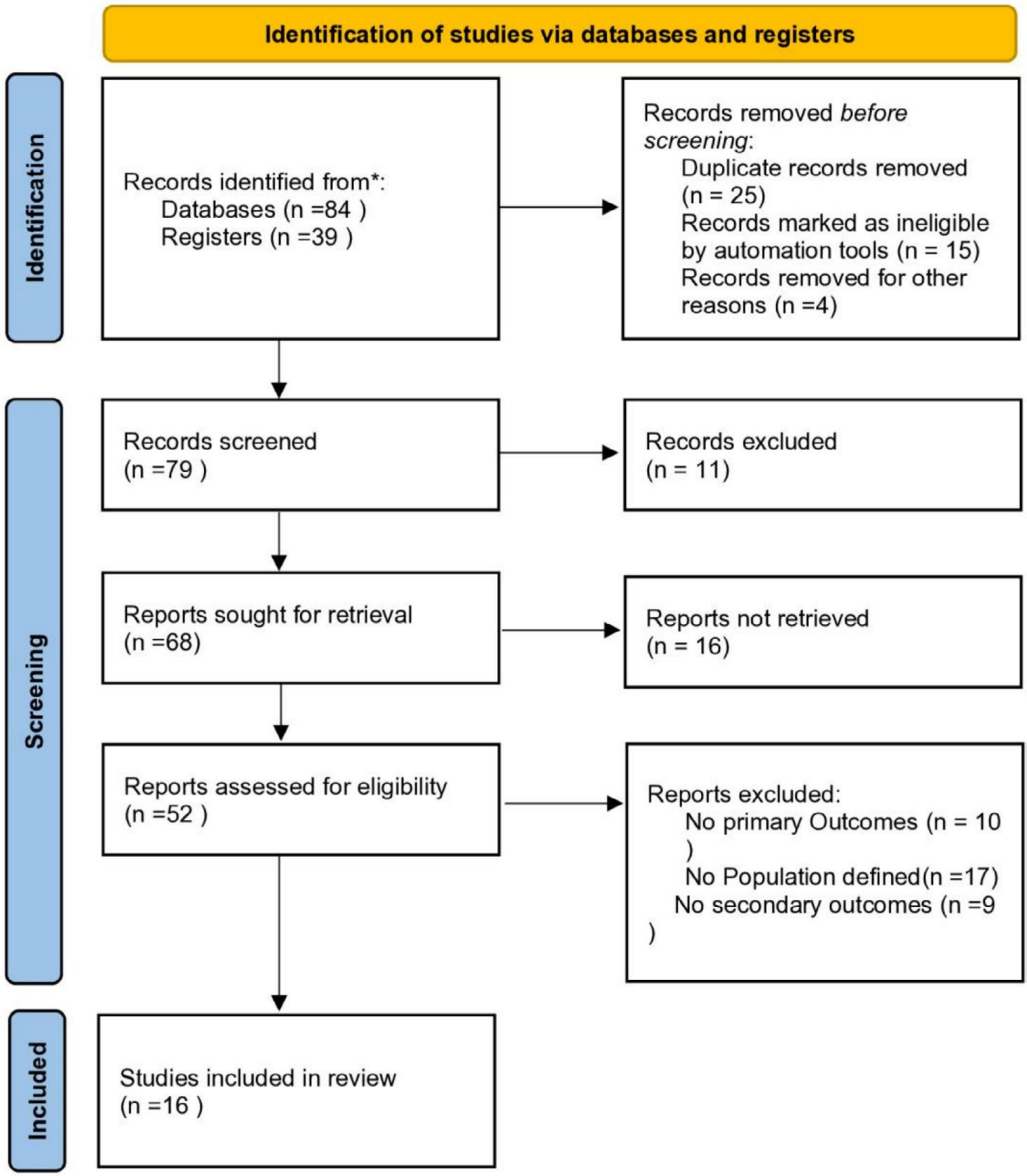
Prisma flowchart.

This study was designed as a systematic review and was conducted in accordance with the Preferred Reporting Items for Systematic Reviews and Meta‐Analyses (PRISMA) guidelines to ensure methodological transparency and reproducibility (Figure 1). The objective of this review was to systematically evaluate the safety and efficacy of LBBAP compared with other pacing strategies, including biventricular pacing (BVP) and His‐bundle pacing (HBP), in patients with heart failure and conduction abnormalities. No quantitative meta‐analysis was performed due to substantial heterogeneity across the included studies.

### 2.2 Search Strategy

A comprehensive literature search was conducted in PubMed, MEDLINE, and Scopus databases to identify relevant studies published up to December 2024. The search strategy employed a combination of Medical Subject Headings (MeSH) and free‐text terms, including “left bundle branch area pacing,” “biventricular pacing,” “His‐bundle pacing,” “cardiac resynchronization therapy,” “left bundle branch block,” “heart failure,” and “safety and efficacy.” In addition, the reference lists of all eligible articles were manually reviewed to ensure complete capture of relevant studies.

### 2.3 Eligibility Criteria

Studies were included if they met the following criteria: (1) involved adult patients with heart failure receiving LBBAP or comparator pacing therapies; (2) reported outcomes related to left ventricular ejection fraction (LVEF), QRS duration (QRSd), hospitalization, complications, or mortality; and (3) were published in English‐language peer‐reviewed journals. Eligible study designs included randomized controlled trials, prospective cohort studies, and observational studies. Studies were excluded if they were case reports, editorials, reviews, animal studies, pediatric studies, or lacked adequate outcome data.

### 2.4 Quality Assessment

The methodological quality of included studies was assessed using validated tools appropriate to study design. Observational and cohort studies were evaluated using the Newcastle–Ottawa Scale (NOS), which assesses selection, comparability, and outcome domains. Randomized controlled trials were assessed using the Cochrane Risk of Bias Tool, evaluating potential bias related to randomization, allocation concealment, blinding, outcome reporting, and attrition. Studies were categorized as having low, high, or unclear risk of bias, and quality assessments were considered when interpreting study findings.

### 2.5 Data Extraction and Narrative Synthesis

Data extraction was performed using a standardized form capturing study characteristics, sample size, pacing modality, comparator group, follow‐up duration, and reported clinical outcomes. Given the substantial heterogeneity among included studies, a narrative qualitative synthesis was undertaken. Outcomes were summarized descriptively, focusing on changes in LVEF, QRS duration, hospitalization rates, procedural complications, and mortality without statistical pooling.

### 2.6 Management of Heterogeneity

Considerable heterogeneity existed among the included studies with respect to study design (randomized vs. observational), patient populations (heart failure severity, LBBB or RBBB), comparator pacing strategies (BVP, HBP, or right ventricular pacing), and follow‐up duration (4.4–24 months). Therefore, findings were stratified narratively based on pacing modality, baseline ventricular function, and conduction pattern where possible. This descriptive stratification allowed contextual interpretation of results while maintaining the integrity of a systematic review approach.

## 3 Results

### 3.1 Study Characteristics

This systematic review included 16 studies, comprising 2 randomized controlled trials and 14 observational studies, published between 2006 and 2024, with a total of 5799 patients. Sample sizes ranged from 11 to 2533 participants, and follow‐up durations varied from 4.4 to 24 months. The included studies demonstrated substantial methodological diversity, encompassing single‐center and multicenter designs and evaluating LBBAP across heterogeneous heart failure populations, pacing indications, comparator strategies, and follow‐up periods.

### 3.2 Efficacy of LBBAP Across Patient Subgroups

Across the included studies, LBBAP consistently improved cardiac function, particularly in patients with heart failure and interventricular conduction delay. The most robust evidence of efficacy was observed in patients with heart failure with reduced ejection fraction (HFrEF) and left bundle branch block (LBBB). Several studies reported significant improvements in LVEF following LBBAP, including a large multicenter cohort by Vijayaraman et al. (2023), which demonstrated an increase in LVEF from 27% ± 6% to 41% ± 13%. Li, Qiu, et al. (2020) similarly reported LVEF improvement to 44.3% ± 8.7% in patients with LBBB. Studies including patients with preserved or mildly reduced LVEF reported more modest functional gains, suggesting that baseline ventricular dysfunction and conduction pattern influence response to LBBAP.

### 3.3 Electrical Synchronization and QRS Duration

Reduction in QRS duration was a consistent finding across studies, reflecting improved electrical synchrony with LBBAP. The magnitude of QRS narrowing was most pronounced in patients with baseline LBBB, where restoration of physiological activation was achievable. Hua et al. (2022) demonstrated a reduction in QRS duration from 172 to 120 ms with LBBAP, compared with a smaller reduction from 165 to 143 ms using BVP. In contrast, studies including patients without classical LBBB or with right bundle branch block (RBBB) showed less consistent reductions, underscoring the subgroup‐specific effectiveness of LBBAP.

### 3.4 Safety Profile and Short‐ to Mid‐Term Outcomes

Overall, LBBAP was associated with low procedural and post‐procedural complication rates, with most adverse events occurring in the early post‐implantation period. Jastrzębski et al. (2022) reported an overall complication rate of 3.43%, including pneumothorax (0.55%) and lead dislodgement (1.5%). Several studies reported no procedure‐related mortality or major surgical complications. However, the majority of included studies had short to intermediate follow‐up durations, limiting assessment of long‐term lead performance, pacing thresholds, arrhythmia burden, and device durability.

### 3.5 Hospitalization and Mortality

Hospitalization rates for heart failure were generally lower in patients receiving LBBAP compared to conventional pacing strategies, particularly BVP. Hua et al. (2022) reported hospitalization rates of 19.05% in the LBBAP group versus 30.00% in the BVP group. Mortality rates across studies were relatively low and comparable between pacing modalities, ranging from 4.76% to 10%. However, mortality outcomes were inconsistently reported and frequently underpowered, necessitating cautious interpretation.

### 3.6 Comparison With Other Pacing Modalities

When compared with BVP and HBP, LBBAP demonstrated superior electrical resynchronization and comparable or improved clinical outcomes, particularly in HFrEF patients with LBBB. In patients without LBBB, benefits were less uniform, reinforcing the need to restrict conclusions to populations with the strongest supporting evidence. A comprehensive summary of study characteristics and outcomes is provided in Table 1.

**TABLE 1:** Summary of studies evaluating LBBAP outcomes in heart failure patients.

Year	Author (reference)	Study design	Sample size	Patients receiving LBBAP therapy	Patients receiving other pacing therapy	EF (%) after LBBAP	QRS duration (ms)	Outcomes (safety and efficacy)	Hospitalization rates (%)	Complications	Mortality rate (%)	Follow‐up
2022	Hua et al. (2022)	Single‐center, non‐randomized, prospective observational study	41	21	20	≥ 50%	LBBAP reduced QRSdfrom 172 ms to 120 ms, compared to a reduction from 165 ms to 143 ms with BIVP	LBBAP and BIVP improved cardiac function, but LBBAP reduced BNP and hospitalizations more effectively. It is a safe, effective option for HF patients	LBBAP = 19.05% BVP = 30.00%	No surgery‐related complications	LBBAP = 4.76% BVP = 5.00%	24 months
2019	Zhang et al. (2019)	Observational study	11	11	—	All 11 patients improved LVEF by ≥ 5%, with 7 showing > 20% LVEF increase and ≥ 15% LVESD reduction	QRSd was shortened by 28.32% ± 6.28%	LBBAP is a viable CRT technique for correcting LBBB, restoring synchrony, and improving symptoms in systolic HF	9.09%	Severe pneumonia and acute HF (1 patient)	No deaths	6.7 ± 3.3 months
2022	Vijayaraman, Cano, et al. (2022)	Retrospective, multicenter, observational cohort study	121	121	—	LVEF improved from 35% ± 9% to 43% ± 12%	significant reduction in QRSd from 154 ± 21 ms to 145 ± 22 ms	LBBAP is a viable option for CRT or ventricular pacing in RBBB, HF, and LV dysfunction	7.44%	6% had worsening LV function, and 3% developed new‐onset atrial fibrillation	6.61%	1 year
2022	Jastrzębski et al. (2022)	Multicentre observational study	2533	2533	—	Not mentioned	—	LBBAP is feasible for bradyarrhythmia and heart failure	N/A	General complications occurred in 3.43% of cases, including pneumothorax (0.55%) and pocket infections (0.51%). Transseptal route complications (3.67%) included LV perforation (3.67%) and lead dislodgement (1.5%)	No deaths	6.4 months
2022	Vijayaraman, Rajakumar, et al. (2022)	Observational study	359	196	163	Paced QRS duration during LBBAP is comparable to HBP	Paced QRS duration during LBBAP was similar to HBP (125 ± 20.2 ms vs. 126 ± 23.5 ms)	No significant differences were found in death or heart failure hospitalization rates between LBBAP and HBP	LBBAP = 10% HBP = 12%	No complications	LBBAP = 10% vs. HBP = 17%	—
2022	Liang et al. (2022)	Prospective cohorts	25	25	—	Improved	Both BVP and LBBP significantly shortened QRSd	LBBP works better than BVP for heart failure patients with LBBB	—	—	—	—
2006	Gasparini et al. (2006)	Randomized Controlled Trial	74	74	—	LV pacing significantly improved LVEF	Reduced	LV pacing is safe, feasible, and improves ejection fraction, with response rates similar to BIV pacing	—	—	—	12 months
2020	Wang et al. (2020)	Prospective cohort study	40	10	30	Improved	QRS duration decreased more in the LBB‐CRT group than in the BIV‐CRT group	LBBAP is a safe and effective way to correct CLBBB and improve heart function, outperforming BIVP in electrical synchrony	—	—	—	6 months
2023	Vijayaraman et al. (2023)	Observational study	1778	797	981	LVEF increased from 27% ± 6% to 41% ± 13%.	Paced QRS duration in LBBAP was significantly narrower than baseline	LBBAP improves outcomes and may be a viable alternative to BVP for CRT patients	Reduced in LBBAP	Reduced in LBBAP	Reduced in LBBAP	—
2024	Liu et al. (2024)	Prospective cohort study	43	43	—	Improved	Narrowed QRS complex	LBB capture sites can impact synchrony and LVEF in heart failure	—	—	—	6‐month
2020	Li, Qiu, et al. (2020)	Prospective, observational, multicentre study	91	37	54	Improved upto 44.3 ± 8.7	Paced QRS duration ≤ 130 m	LBBAP is a safe, effective alternative to BVP for heart failure with LBBB.	N/A	N/A	N/A	6‐month
2020	Cheng et al. (2020)	Single‐centre, randomized controlled non‐inferiority trial	180	90	90	LVEF improvement in the LBBAP group was 4.36 times greater	—	LBBAP ensure safety and improve synchrony, LVEF, and outcomes	—	—	—	18 month
2021	Vijayaraman et al. (2021)	Retrospective, multicenter, observational cohort study	325	325	—	LVEF improved from 33% ± 10% to 44 ± 11	With LBBAP, QRSd reduced from 156 ± 20 ms to 150 ± 24 ms.	LBBAP is a safe and feasible alternative for CRT.	(8%)	Three patients (3%) develop AF	7.5%	13 ± 8 months
2023	Sussenbek et al. (2023)	Retrospective observational study	80	Not specified	Not specified	Improved	QRSd did not differ significantly between LBBAP and Biv CRT	LBBAP and BVP CRT reduced QRSd from 172 to 152 ms.	NA	NA	NA	—
2022	Jiang et al. (2022)	Single‐center retrospective study	36	—	—	36 LVEF ≤ 35% (*n* = 15). LVEF > 35% (*n* = 21)	LBBAP shortens QRSd and improves cardiac function in LBBB patients with LVEF ≤ 35%	LBBAP is effective for preserving cardiac function in early heart failure with LBBB and LVEF > 35%	Higher in patients with LVEF ≤ 35% than those with LVEF > 35% (27.3% vs. 17.6%)	Pocket infection in one patient	Higher in patients with LVEF ≤ 35% than those with LVEF > 35% (18.2% vs. 0%).	6–12 months
2021	Liu, Hu, et al. (2021)	Multicenter, prospective cohort study	62	27	35	QRSd was significantly shortened after LBBAP	Improved	LBBAP enhances cardiac function, synchronization, and efficiency	N/A	N/A	N/A	4.4 ± 1.4 months

## 4 Discussion

### 4.1 Introduction to LBBAP in Heart Failure

Heart failure (HF) with reduced ejection fraction (HFrEF) presents significant morbidity and mortality, often linked to dyssynchronous ventricular contraction caused by left bundle branch block (LBBB) (Friedman et al. 2021). Traditional cardiac resynchronization therapy (CRT) with biventricular pacing (BVP) or right ventricular pacing (RVP) has shown limited efficacy in certain patients due to their inability to restore physiological conduction (Mirmaksudov et al. 2024). LBBAP, as a novel physiological pacing method, directly targets the left conduction system, aiming to restore both electrical and mechanical synchrony (Padala and Ellenbogen 2020). This discussion synthesizes findings from 16 studies, highlighting the safety, efficacy, and potential advantages of LBBAP over conventional pacing strategies in HF patients.

### 4.2 Efficacy of LBBAP: Improvements in LVEF and QRS Duration

One of the most significant findings across the studies reviewed is the improvement in left ventricular ejection fraction (LVEF) and QRS duration (QRSd) following LBBAP. Studies, such as those by Zhang et al. (2019) and Vijayaraman et al. (2023), reported substantial LVEF improvements from baseline. For instance, in one study, LVEF increased from 27% ± 6% to 41% ± 13%, demonstrating LBBAP's ability to address systolic dysfunction effectively. Similarly, reductions in QRSd—critical for achieving electrical synchrony—were consistently observed.

This improvement in QRS narrowing is particularly important, as prolonged QRS duration is associated with worse outcomes in HF (Kashani and Barold 2005). The ability of LBBAP to achieve physiological pacing results in more efficient ventricular contraction and reduced electrical dyssynchrony compared to BVP or RVP (Mirmaksudov et al. 2024), as highlighted in studies like those by Hua et al. (2022) and Jiang et al. (2022).

### 4.3 Comparison With Traditional Pacing Methods

LBBAP offers several advantages over traditional pacing methods, such as BVP and RVP. BVP, though effective in many cases, does not always restore physiological activation patterns (Castagno et al. 2024). Conversely, RVP is often associated with pacing‐induced cardiomyopathy due to dyssynchronous activation (Khurshid and Frankel 2023). LBBAP, by directly engaging the His‐Purkinje system, provides a more natural depolarization pathway (Bressi et al. 2023).

For example, studies by Gasparini et al. (2006) and Li, Qiu, et al. (2020) demonstrated that LBBAP not only improved electrical synchronization but also showed superior mechanical efficiency compared to BVP. The reduction in left ventricular end‐systolic diameter (LVESD) and improvement in LVEF underscores LBBAP's potential to mitigate HF progression more effectively than conventional pacing (Chen et al. 2022).

Additionally, studies such as those by Wang et al. (2020) revealed that LBBAP outperformed BVP in achieving electrical synchrony, which is crucial for optimizing cardiac output. The safety profile of LBBAP was also highlighted, with fewer device‐related complications reported compared to BVP and RVP.

### 4.4 Safety and Patient Outcomes

Safety is a critical consideration in selecting a pacing strategy for HF patients. LBBAP has been shown to be a feasible and well‐tolerated approach across various studies (Li, Yan, et al. 2020). Adverse events, such as lead dislodgement, device infection, and pacing‐induced arrhythmias, were infrequent (Vouliotis et al. 2023). Notably, studies like those by Vijayaraman, Cano, et al. (2022) and Liu et al. (2024) reported fewer HF exacerbations in patients receiving LBBAP compared to those with conventional pacing.

Moreover, the ability of LBBAP to maintain electrical synchrony over time, without causing dyssynchrony‐related complications, makes it a safer alternative. Reduced arrhythmic events and better long‐term outcomes further support its use in clinical practice (Diaz et al. 2023).

### 4.5 Advantages in Specific Patient Populations

LBBAP may be particularly advantageous in certain subgroups of HF patients. For instance, in those with narrow or borderline QRS complexes who do not meet traditional criteria for CRT, LBBAP has shown potential for improving cardiac function (Bleeker et al. 2006). Additionally, studies by Sussenbek et al. (2023) suggest that patients with RBBB or atypical conduction patterns may also benefit from LBBAP, given its ability to achieve more uniform ventricular activation.

In early‐stage HF with preserved LVEF (> 35%), LBBAP has demonstrated benefits in preserving cardiac function and delaying HF progression (Zeng et al. 2023), as highlighted in the work of Jiang et al. (2022). These findings broaden the applicability of LBBAP beyond conventional CRT indications.

### 4.6 Limitations

Despite its promising outcomes, LBBAP is not without limitations. Most studies included in this review were observational or retrospective, which limits the ability to establish causality. There was significant heterogeneity in study designs, patient populations, pacing techniques, and outcome measures, making direct comparisons challenging.

The lack of long‐term follow‐up in many studies raises questions about the durability of LBBAP's effects. While short‐term improvements in LVEF and QRSd are promising, data on long‐term survival, HF progression, and potential late complications are limited. Standardization of LBBAP techniques, including lead positioning and pacing parameters, is also lacking, which may impact outcomes.

### 4.7 Future Directions

To solidify the role of LBBAP in HF management, future research should focus on randomized controlled trials with larger sample sizes and longer follow‐up durations. These studies should aim to compare LBBAP directly with BVP and RVP in diverse HF populations, including those with varying degrees of systolic dysfunction and conduction abnormalities (El Iskandarani et al. 2024).

Additionally, exploring the impact of LBBAP on other outcomes, such as quality of life, functional capacity, and healthcare utilization, will provide a more comprehensive understanding of its benefits. Further studies are also needed to identify optimal patient selection criteria and refine pacing protocols for LBBAP (Tan et al. 2021).

## 5 Conclusion

The findings of this systematic review underscore the potential of LBBAP as a transformative pacing strategy for heart failure patients, particularly those with LVEF ≤ 50% and LBBB. By directly targeting the left conduction system, LBBAP offers superior electrical and mechanical synchronization, resulting in significant improvements in LVEF, QRS duration, and clinical outcomes (Cano et al. 2023).

While the existing evidence is promising, limitations such as heterogeneity in study designs, lack of standardized protocols, and short follow‐up durations highlight the need for further research (Linden and Hönekopp 2021). As the field evolves, LBBAP has the potential to become a standard therapy for HF, offering hope for improved outcomes in a challenging patient population (Glikson et al. 2023).

## Author Contributions

Hina Ahmed Siddiqui: writing and supervision. Mounika Kotte: conceptualization, methodology, writing. Hilana Soliman Omar: formal analysis, data correction. Saad Manzoor: project administration, writing, revision. Zohaib Qasim: writing, validation, software, investigation. Fnu Abdullah: supervision, methodology, writing. Sadia Siddique: project administration, writing, revision. Muhammad Bari Hassan: investigation, software, resources, revision. Ali Karim: writing, methodology. Hansa Devi: software, supervision. Ekta Rani: writing, literature search, revision. Aniket Tara: resources, writing, methodology. Payal Bai: supervision, writing, revision, methodology. Sajid Ali: writing, methodology, software. Hina Kumari: resources, writing, methodology. Sitara Jabeen: writing, software, project administration. Vikram Kumar: supervision, resources, writing. Abida Perveen: supervision, writing, revision, methodology, software.

## Funding

The authors received no specific funding.

## Conflicts of Interest

The authors declare no conflicts of interest.

## Data Availability

Data sharing not applicable to this article as no datasets were generated or analyzed during the current study.
